# Incidence and diagnosis of Acute kidney injury in hospitalized adult patients: a retrospective observational study in a tertiary teaching Hospital in Southeast China

**DOI:** 10.1186/s12882-017-0622-6

**Published:** 2017-06-24

**Authors:** Xiaoyan Cheng, Buyun Wu, Yun Liu, Huijuan Mao, Changying Xing

**Affiliations:** 1 Department of Nephrology, The First Affiliated Hospital of Nanjing Medical University, Nanjing, 210029 China; 2 Intensive Care Unit, Sichuan Cancer Hospital & Institute, Sichuan Cancer Center, School of Medicine, University of Electronic Science and Technology of China, Chengdu, 610041 China; 3 Department of Information, The First Affiliated Hospital of Nanjing Medical University, Nanjing, 210029 China

**Keywords:** Acute kidney injury, Diagnosis, Incidence, Prognosis, Risk factors

## Abstract

**Background:**

Acute kidney injury (AKI) places a heavy burden on the healthcare system in China and is usually misdiagnosed. However, there are limited studies that have described the epidemiology and diagnosis of AKI in China. The aim of this study was to describe the incidence and diagnosis of AKI in hospitalized adult patients in a tertiary teaching hospital in southeast China.

**Methods:**

All adult patients hospitalized from October 1, 2013 to September 30, 2014 in the First Affiliated Hospital of Nanjing Medical University were screened using the Lab Administration Network. AKI definition and staging were based on the KDIGO AKI criteria. Demographic characteristics, laboratory examination, clinical data, and clinical outcomes of AKI patients were recorded and analyzed.

**Results:**

The incidence of AKI was 1.6% (1401/87196). The 30-day mortality was 35.3%. AKI stage 1, 2, 3 and RRT accounted for 38.0% (532/1401), 22.0% (309/1401), 40.0% (560/1401), and 16.3% (228/1401) of patients, respectively. The Renal, other Internal Medicine, Surgery, and ICU Departments accounted for 7.4%, 37.1%, 30.1%, and 25.4% of AKI patients, respectively. The timely diagnosis rate, delayed diagnosis rate, and missed diagnosis rate were 44% (616/1401), 3.3% (46/1401), and 52.7% (739/1401), respectively. Patients hospitalized in the Renal Department had the highest AKI diagnosis rate (89.3%, 88/103), while missed diagnosis rate of the surgical patients was as high as 75.1% (317/422). Multivariable logistic regression analysis indicated that presence of tumors, higher serum albumin, and AKI stage 1 were associated with failure to timely diagnose AKI, whereas presence of chronic kidney disease, oliguria, higher blood urea nitrogen, and greater number of organ failures correlated with earlier diagnosis.

**Conclusions:**

AKI was characterized by a high incidence, high short-term mortality, and high missed diagnosis rate in hospitalized adult patients in our hospital. Interventions for improving diagnosis of AKI are urgently needed.

**Electronic supplementary material:**

The online version of this article (doi:10.1186/s12882-017-0622-6) contains supplementary material, which is available to authorized users.

## Background

Acute kidney injury (AKI) is a common complication in hospitalized patients that is characterized by a high incidence, high mortality, poor renal prognosis, and increased medical costs [1–3]. There were about 13.3 million cases of AKI reported worldwide in 2013 with about 85% of cases occurring in low and middle income countries [4]. In China, the largest developing country in the world with about 20% of the global population and unbalanced development across regions, AKI occurred in 1.0%–10.7% of hospitalized patients and thus placed a heavy burden on the healthcare system in China as well as all other countries [5, 6].

With the deepening recognition of the great burden to society and the pathogenesis of AKI, the International Society of Nephrology launched a global target of “0 by 25”—no patient deaths due to untreated AKI by 2025—to improve the diagnosis and treatment of AKI [7]. The effective treatment of AKI is strongly dependent on a timely diagnosis. However, the mean missed diagnosis rate of AKI is reportedly as high as 74.2% [5], which may increase mortality and retard the full implementation of the global target of “0 by 25”. As the largest developing and most populated country, the financial status and medical availability in China are quite different among regions. Thus, reports on the epidemiology and diagnosis of AKI remain deficient [5, 6, 8–12].

Therefore, the aim of this study was to investigate the incidence and diagnosis of AKI in hospitalized adult patients from a tertiary teaching hospital located in a southeast coastal economically developed area in China. Particular attention was focused on the diagnosis of AKI and the characteristics of the population underdiagnosed.

## Methods

### Study population

This was a retrospective observational study. All patients admitted to the First Affiliated Hospital of Nanjing Medical University (Nanjing, China) from October 1, 2013 to September 30, 2014 were screened using the Lab Administration Network. The First Affiliated Hospital is one of the largest medical centers in Jiangsu Province with 51 departments (not including the departments of pediatrics and gynecology/obstetrics). The hospital has more than 3000 beds and the annual number of hospitalized patients was nearly 140,000 in 2016. The Ethics Committee of the hospital waived the requirement for written consent because of its non-interventional study. All patients aged >18 years who underwent at least two serum creatinine (SCr) examinations during hospitalization were enrolled. Patients on regular dialysis, or received kidney transplantation, or hospitalized for less than 24 h were excluded. This study adhered to the STROBE guidelines on reporting observational studies.

### Data source

The hospitalization records of all potential study participants were reviewed. If the AKI criteria were met, the following information was recorded: age, sex, department residence, previous disease history, lab examination results, baseline SCr, SCr at diagnosis of AKI, primary cause of AKI (surgery, hypovolemia, nephrotoxic drugs, sepsis, etc.), diagnosis of AKI (timely, delayed, missed), oliguria or not, estimated glomerular filtration rate (eGFR) on admission and discharge (calculated according to the Chronic Kidney Disease Epidemiology Collaboration formula), number of extra-renal organ failures, renal replacement therapy (RRT) or not, length of hospital stay, length of intensive care unit (ICU) stay, total medical costs, patients prognosis on day 30 after AKI, and renal prognosis upon discharge. The etiological diagnosis of each AKI patient was conducted by two nephrologists. In case of uncertainty or doubt, the etiological diagnosis was discussed with the director.

### Definition

The lowest SCr value during hospitalization was defined as baseline SCr. The definition and staging of AKI were based on the creatinine criteria of KDIGO AKI criteria [13]: i.e., SCr increase of more than 26.5 μmol/L in 48 h or 50% higher than upon admission during 7 days.

According to the KDIGO criteria, stage 1 encompasses a SCr level increase of ≥0.3 mg/dL (26.5 μmol/L) within 48 h or increase in SCr to ≥1.5 times baseline, which is known or presumed to have occurred within 7 days; stage 2, increase in SCr to 2.0–2.9 times baseline; stage 3, increase in SCr to 3.0 times baseline or to ≥4.0 mg/dL (353.6 μmol/L), or receipt of renal replacement therapy (RRT).

CKD is a condition in which the kidneys have been damaged and have not worked normally for at least 3 months.

### Definition of outcomes

Patient outcomes included 30-day mortality, renal recovery, length of hospitalization, and cost of hospitalization.

Timely diagnosis was defined as recognition of AKI by the physicians in charge within 3 days of the point from which AKI could be diagnosed, otherwise diagnosis was defined as delayed, and unrecognized was defined as a missed diagnosis [5].

Renal prognosis was classified as complete recovery, partial recovery, or loss of renal function based on the SCr level at discharge compared to that at baseline. Complete recovery of kidney function was defined as a SCr level of no more than 0.5 mg/dL (44 μmol/L) greater than the baseline value. Partial recovery was limited to the patients not dependent on dialysis with a SCr level between 0.5 mg/dL greater than the baseline value and the highest SCr value during hospitalization. Loss of renal function was defined as a continuously increasing SCr value or the need for RRT [14].

### Statistical analysis

Continuous data were presented as the mean ± standard deviation or median with interquartile range and categorical variables as number and percentage. Comparisons among groups were made by one-way analysis of variance or the Kruskal–Wallis test for continuous variables, or the χ^2^ test for categorical variables. To explore the potential risk factors for prognosis (30-day mortality and loss of renal function) and failure to diagnose AKI, univariate logistic regression analyses were performed, and the variables that were found to be statistically significant (*P* < 0.05) in the univariate analysis were included in multivariate analyses with the stepwise selection. A two-sided probability (*p*) value of <0.05 was deemed significant. All statistical analyses were performed using SAS software (version 9.2; SAS Institute, Cary, NC, USA).

## Results

### Incidence of AKI

**Fig. 1:**
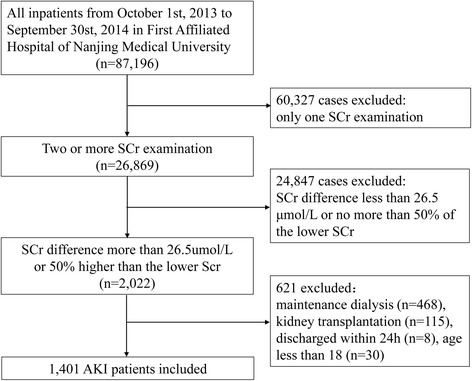
The study flow

A total of 87,196 patients admitted to the First Affiliated Hospital of Nanjing Medical University from October 1, 2013 to September 30, 2014 were initially screened for inclusion. Of these patients, 26,869 (30.8%) met the criteria of two or more SCr values. A total of 621 cases were excluded: 468 with regular dialysis, 115 with kidney transplantation, 30 aged less than 18 years, and eight hospitalized for less than 24 h. Finally, 1401 cases (1.61%) in this cohort were diagnosed with AKI (Fig. 1).

The incidence of AKI in the Nephrology Department was 3.5% (103/2943) and the incidence in the other Internal Medicine, Surgery and ICU Departments were 1.3% (520/40052), 1.0% (422/42223), and 18.0% (356/1978), respectively.

### Characteristics of AKI patients

The average age of AKI patients was 63.2 ± 37.2 years and the ratio of males to females was 2.0:1. Urban residents accounted for 58.3% of AKI cases. The average admission eGFR was 67.1 ± 37.2 mL/min/1.73 m^2^, the median hospital stay was 18 days, and the median cost was 57,200 Chinese yuan (CNY). The percentages of patients with a history of smoking and drinking were 21.0% and 13.1%, respectively. The prevalence of associated comorbidities was as follows: cardiovascular disease, 58.9%; chronic kidney disease (CKD), 8.3%; diabetes, 17.9%; chronic liver disease, 7.6%; chronic lung disease, 6.5%; rheumatic autoimmune disease, 4.1%; and malignancy, 29.6%. During hospitalization, the percentages of AKI patients receiving diuretics, nephrotoxic antibiotics, or contrast agent were 32.2%, 29.8%, and 14.7%, respectively.

Of all the AKI patients, 7.4% were from the Nephrology Department, 37.1% from other Internal medicine Departments, 30.1% from the Department of Surgery, and 25.4% from the ICU (Table 1). Among AKI patients from other Internal medicine departments, 26.4% (137/520) were from the Cardiovascular Department and 19.4% (101/520) were from the Hematology Department. Among AKI patients from the Department of Surgery, 40.3% (170/422) were from the Cardiac Surgery Department, 25.4% (107/422) from the Urology Department, and 18.2% (77/422) from the Hepatic Surgery Department.

**Table 1:** Comparison of AKI patients’ characteristics in different departments

Department	Total	ICU	Other Internal	Surgery	Nephrology	*P* value
	(*n* = 1401)	(*n* = 356)	(*n* = 520)	(*n* = 422)	(*n* = 103)	
Age (years)	63.2 ± 37.2	64.4 ± 17.7	66.2 ± 17.9	59.7 ± 14.5	57.7 ± 18.4	<0.001*
Male (%)	939 (67.0)	249 (69.9)	352 (67.7)	282 (66.8)	56 (54.4)	0.030*
City (%)	816 (62.0)	214 (65.0)	363 (71.7)	191 (50.1)	48 (47.5)	<0.001*
Admission eGFR (%)						<0.001*
≥ 90 (mL/min/1.73 m^2^)	461 (32.9)	117 (32.8)	155 (29.8)	184 (43.6)	5 (4.8)	
60–89 (mL/min/1.73 m^2^)	357 (25.5)	86 (24.2)	134 (25.8)	130 (30.8)	7 (6.8)	
30–59 (mL/min/1.73 m^2^)	266 (19.0)	75 (21.1)	123 (23.6)	55 (13.0)	13 (12.6)	
< 30 (mL/min/1.73 m^2^)	317 (22.6)	78 (21.9)	108 (20.8)	53 (12.6)	78 (75.7)	
Comorbidities (%)						
Cardiovascular disease	825 (58.9)	234 (65.7)	307 (59.0)	249 (59.0)	35 (34.0)	<0.001*
Diabetes	251 (17.9)	68 (19.1)	117 (22.5)	57 (13.5)	9 (8.7)	<0.001*
Pre-existing CKD	116 (8.3)	25 (7.0)	50 (9.6)	24 (5.7)	17 (16.5)	0.002*
Chronic lung disease	91 (6.5)	30 (8.4)	36 (6.9)	11 (2.6)	14 (13.6)	<0.001*
Chronic liver disease	106 (7.6)	28 (7.9)	47 (9.0)	30 (7.1)	1 (1.0)	0.042*
Malignancy	415 (29.6)	67 (18.8)	201 (38.7)	133 (31.5)	14 (13.6)	<0.001*
Susceptible risk factors (%)						
Using diuretics	451 (32.2)	145 (40.7)	175 (33.7)	114 (27.0)	17 (16.5)	<0.001*
Using nephrotoxic antibiotics	418 (29.8)	154 (43.3)	207 (39.8)	41 (9.7)	16 (15.5)	<0.001*
Using contrast Agent	206 (14.7)	33 (9.3)	87 (16.7)	83 (19.7)	3 (2.9)	<0.001*
Hypovolemia	521 (37.2)	220 (62.0)	112 (21.5)	182 (43.1)	7 (6.8)	<0.001*
Operation	514 (36.7)	119 (33.4)	46 (8.8)	346 (82.0)	3 (2.9)	<0.001*
Sepsis	154 (11.0)	78 (21.9)	49 (9.4)	21 (5.0)	6 (5.8)	<0.001*
Oliguria (%)	300 (21.4)	121 (34.0)	89 (17.1)	43 (10.2)	47 (45.6)	<0.001*
Extra-renal organs failure	674 (48.1)	254 (71.3)	329 (63.3)	188 (44.5)	25 (24.2)	<0.001*
AKI Stage (%)						<0.001*
1	532 (38.0)	107 (20.1)	224 (42.1)	189 (35.5)	12 (2.3)	
2	309 (22.1)	67 (21.7)	120 (38.8)	112 (36.2)	10 (3.2)	
3	560 (40.0)	182 (32.5)	176 (31.4)	121 (21.6)	81 (14.5)	
AKI classification (%)						0.068
Pre-renal	589 (42.1)	121 (20.5)	224 (38.0)	239 (40.6)	5 (0.8)	
Renal	731 (52.2)	229 (31.3)	279 (38.1)	136 (18.6)	88 (12.0)	
Post-renal	80 (5.7)	6 (7.5)	17 (21.2)	47 (58.8)	10 (12.5)	
Interval between admission and AKI diagnosed (days)	5 (1,11)	4 (1,11)	5 (1,12)	7 (3,12)	1 (0,2)	<0.001*
Diagnosis (%)						
Timely	616 (44.0)	192 (54.2)	243 (46.7)	92 (21.8)	88 (85.4)	<0.001*
Delayed	46 (3.3)	14 (3.9)	15 (2.9)	13 (3.1)	4 (3.9)	0.008*
Missed	739 (52.7)	149 (41.9)	262 (50.4)	317 (75.1)	11 (10.7)	<0.001*
Only 2 SCr detection (%)	59 (4.2)	8 (2.25)	36 (6.9)	14 (3.3)	1 (1.0)	<0.001*
Renal prognosis (%)						<0.001*
Complete recovery	628 (44.8)	132 (37.1)	193 (37.1)	274 (64.9)	29 (28.2)	
Partial recovery	178 (12.7)	28 (7.9)	76 (14.6)	50 (11.8)	24 (23.3)	
Loss of renal function	321 (22.9)	122 (34.3)	112 (21.5)	44 (10.4)	43 (41.7)	
No data^a^	274 (19.6)	74 (20.8)	139 (26.7)	54 (12.8)	7 (6.8)	
RRT dependence	228 (16.3)	88 (24.7)	46 (8.8)	34 (8.1)	60 (58.3)	<0.001*
ICU stay (days)	0 (0–5)	11 (5–21)	0 (0–0)	1 (0–2)	0 (0–0)	<0.001*
Hospital stay (days)	22.3 ± 19.8	23.6 ± 22.2	19.6 ± 17.7	26.0 ± 21.1	17.3 ± 9.9	<0.001*
Costs (thousand CNY)	57.2 (25.0–121.6)	106.6 (50.9–199.1)	34.3 (15.0–64.8)	80.6 (39.3–134.6)	23.8 (14.7–37.0)	<0.001*
30-day mortality	495 (35.3%)	227 (63.8%)	204 (39.2%)	51 (12.1%)	13 (12.6%)	<0.001*

^a^referred to no SCr detection after diagnosis of acute kidney injury. *: *P*﻿ < 0.05.

*AKI* acute kidney injury, *CKD* chronic kidney disease, *CNY* Chinese yuan, *eGFR* estimated glomerular filtration rate, *ICU* intensive care unit, *RRT* renal replacement therapy

### Diagnosis rate of AKI inpatients

The incidence of AKI was 1.6% (1401/87196), while detection rate of AKI in hospitalized patients was 0.76% (662/87196). The timely diagnosis rate and delayed diagnosis rate were 44.0% (616/1401) and 3.3% (46/1401), respectively. The missed diagnosis rate was as high as 52.7% (729/1401).

The timely diagnosis rate of AKI differed among departments. The Nephrology Department was the highest (89.3%), followed by the ICU (58.1%), other Internal Medicine Departments (49.6%), and the Surgery Department (24.9%). Parenchymal renal injury was diagnosed in 52.2% of AKI patients, post-renal injury in 5.7%, and pre-renal injury in 42.1% (Table 2). The diagnosis rates of stages 1, 2, and 3 AKI were 22.6%, 29.1% and 80.7%, respectively (Table 3).

**Table 2:** Comparison on diagnosis and prognosis in different etiology of AKI

Variables	Pre-Renal (*n* = 589)	Parenchymal Renal Injury (*n* = 732)	Post-Renal (*n* = 80)	*P* Value
Age (years)	61.1 ± 16.7	64.8 ± 17.6	63.6 ± 15.1	<0.001*
Male (%)	389 (66.0)	493 (67.3)	57 (71.3)	0.626
eGFR at admission (mL/min/1.73 m2)	88.8 ± 26.2	52.9 ± 36.0	40.6 ± 37.0	<0.001*
Interval Between Admission and AKI Diagnosed (days)	7 (2,12)	4 (1,10)	1 (1,8)	<0.001*
Diagnosis (%)				<0.001*
Timely	78 (13.2)	495 (67.6)	43 (53.8)	
Delayed	6 (1.0)	37 (5.1)	3 (3.8)	
Missed	505 (85.7)	200 (27.3)	34 (42.5)	
Only 2 SCr detection (%)	37 (6.3)	19 (2.6)	3 (3.7)	0.004*
Renal prognosis (%)				<0.001*
Completely recovery	390 (66.2)	194 (26.5)	44 (55.0)	
Partial recovery	34 (5.8)	137 (18.7)	7 (8.7)	
Loss of renal function	15 (2.6)	289 (39.5)	17 (21.2)	
No data^a^	150 (25.5)	112 (15.3)	12 (15.0)	
ICU stay (days)	0 (0–3)	0 (0–8)	0 (0–0)	<0.001*
Hospital stay (days)	22.8 ± 19.0	22.4 ± 20.7	18.9 ± 16.8	0.009*
Hospital costs (thousand CNY)	67.4(31.7–123.0)	51.5 (24.0–128.7)	21.1 (11.6–49.3)	<0.001*
30-day Mortality (%)	141 (23.9)	339 (46.3)	15 (18.8)	<0.001*

^a^referred to no SCr detection after diagnosis of acute kidney injury. *: *P* < 0.05.

*CNY* Chinese yuan, *eGFR* estimated glomerular filtration rate, *ICU* intensive care unit

**Table 3:** Comparison on diagnosis and prognosis of AKI different stages

Variables	Stage 1 (*n* = 532)	Stage 2 (*n* = 309)	Stage 3 (*n* = 560)	*P* value
Age (years)	63.5 ± 16.6	61.7 ± 17.9	63.6 ± 17.3	0.292
Male (%)	375 (70.5)	309 (64.7)	364 (65.0)	0.097
eGFR (mL/min/1.73 m2)	76.9 ± 31.9	81.4 ± 29.4	50.4 ± 39.3	<0.001*
Interval between admission and AKI diagnosed (days)	6 (2,11)	7 (3,14)	3 (1,9)	<0.001*
Diagnosis (%)				<0.001*
Timely	110 (20.7)	76 (24.6)	430 (76.8)	
Delayed	10 (1.9)	14 (4.5)	22 (3.9)	
Missed	412 (77.4)	219 (70.9)	108 (19.3)	
Only 2 SCr detection (%)	38 (7.1)	10 (3.2)	11 (2.0)	<0.001*
Renal prognosis at discharge (%)				<0.001*
Completely recovery	311 (58.5)	171 (55.3)	146 (26.1)	
Partial recovery	51 (9.6)	42 (13.6)	85 (15.2)	
Loss of renal function	38 (7.1)	38 (12.3)	245 (43.7)	
No data^a^	132 (24.8)	58 (18.8)	84 (15.0)	
ICU stay (days)	0 (0–2)	0 (0–4)	0 (0–9)	<0.001*
Hospital stay (days)	20.9 ± 18.1	24.3 ± 17.7	22.6 ± 22.2	<0.001*
Hospital costs (thousand CNY)	53.5 (20.8–103.5)	68.9 (33.4–132.9)	54.4 (24.8–132.4)	<0.001*
30-day mortality (%)	121 (22.7)	105 (34.0)	269 (48.0)	<0.0001*

^a^referred to no SCr detection after diagnosis of acute kidney injury.*: *P*﻿ < 0.05.

*CNY* Chinese yuan, *eGFR* estimated glomerular filtration rate, *ICU* intensive care unit

### Risk factors for 30-day mortality, loss of renal function, and failure to timely diagnosis in AKI patients

Univariate logistic regression analysis was used to screen for independent variables as potential risk factors of 30-day mortality (Additional file 1: Table S1) or loss of renal function (Additional file 1: Table S3). The significant variables were further included in a multivariable logistic backward regression model. The results indicated that age, presence of chronic liver disease, oliguria, extra-renal organ failure, malignancy, and chronic pulmonary diseases were independent risk factors for 30-day mortality in hospitalized patients with AKI (Additional file 1: Table S2), and that age, presence of extra-renal organ failure, higher AKI stage, presence of oliguria, receiving RRT, no prior surgery, higher total bilirubin, and lower hemoglobin and lower serum albumin were independent risk factors for loss of renal function (Additional file 1: Table S4). Univariate (Additional file 1: Table S5) and crude multivariate (Table 4) logistic regression analysis showed that presence of tumors, higher serum albumin, and AKI stage 1 were associated with failure to timely diagnose AKI, while presence of CKD, presence of oliguria, higher blood urea nitrogen, and greater number of organ failures correlated with timely diagnosis.

**Table 4:** Crude multivariable logistic regression analysis on failure to timely diagnose AKI

Variable	Failure to timely diagnose AKI		
	*P* value	OR	95% CI
Chronic kidney disease (yes: no)	<0.001	0.27	0.16–0.45
Presence of tumor (yes: no)	0.032	1.47	1.03–2.00
AKI Stage 2: stage 1	0.874	0.97	0.67–1.41
AKI Stage 3: stage 1	<0.001	0.24	0.17–0.36
Presence of oliguria (yes: no)	0.006	0.51	0.32–0.82
Receiving renal replacement therapy (yes: no)	<0.001	0.06	0.02–0.14
Extra-Renal Organ Failure (yes: no)	<0.001	0.63	0.53–0.76
Serum albumin (per 1 g/L)	<0.001	1.04	1.11–1.06
Blood urea nitrogen (per 1 mmol/L)	<0.0001	0.95	0.93–0.96
C statistic (95% CI)	0.88 (0.86–0.90)		
Test of Goodness of Fit	χ^2^ = 10.88, *P* = 0.208		

*AKI* acute kidney injury, Variables included in the multivariate logistic regression comes from variables that were found to be statistically significant (*P* < 0.05) in the univariate analysis (see in Additional file 1: Table S5)

## Discussion

In this retrospective study conducted in a tertiary teaching hospital in southeast China, the incidence of AKI was 1.6% in hospitalized patients with a 30-day mortality of 35.3%. The timely diagnosis rate, delayed diagnosis rate, and missed diagnosis rate were 44.0%, 3.3%, and 52.7%, respectively. Furthermore, the presence of tumors, higher serum albumin, and AKI stage 1 were associated with failure to timely diagnose AKI, while CKD, presence of oliguria, higher blood urea nitrogen, greater number of organ failures correlated with timely diagnosis.

AKI is a heavy global burden that is associated with both short-term and long-term mortality. Although the prognoses of AKI patients were poor, AKI has been recently considered as a preventable and treatable disease [4, 15]. In 2013, the International Society of Nephrology launched a global target of “0 by 25” with a goal of no AKI patient deaths due to untreated acute kidney failure by 2025 [15]. Therefore, exact and sufficient data of epidemiology is a crucial first-step to reduce morbidity and mortality of AKI patients, especially in developing countries, which represent 85% of the world population [2]. The incidence of AKI was reportedly about 20% in high-income countries [3, 15], which varied from 1.0% to 10.7% in China [5, 6, 8, 16, 17]. In this study, the incidence of AKI was 1.6%, similar to the figure reported by Yang et al. [5] and much less than that reported by Xu et al. [6]. In addition, given that AKI usually occurred in critically ill patients, and patients receiving major surgery or complicating with a variety of chronic diseases, thus AKI occurred mainly in ICU (25.4%), surgery (30.1%) and other internal medicine departments (37.1%). In contrast, patients with AKI attributing to primary or secondary glomerulonephritis may be admitted to Nephrology Department. Therefore, the smallest caseload of AKI (7.4%) was managed directly in Nephrology Department, while much more AKI patients hospitalized in different departments all around the hospital. The findings of this study provide additional evidence of the epidemiology of AKI in China.

Despite the high incidence, AKI in hospitalized patients is characterized by a high rate of missed diagnosis. The missed diagnosis rate was about 57% even in high-income countries [18]. However, the missed diagnosis rate was greater in China. A survey of 44 hospitals in 22 cities in China showed that diagnosis was missed in 74.2% of AKI inpatients, while timely diagnosis rate was only 21.2% and delayed rate was 4.6% [5]. The results of this study showed that the timely diagnosis rate was 44.0% and the missed diagnosis rate was 52.7%. These results revealed that our hospital in a developed area of China had a higher timely diagnosis rate than the average rate in China [5] and a similar diagnosis rate to high-income countries [18], although the timely diagnosis rate was still unsatisfactory. Thus, a present challenge is how to decrease the missed diagnosis rate of AKI in hospitalized patients.

Identifying hospitalized patients with missed diagnosis may help to develop the strategies to reduce the missed diagnosis rate in the future. This study showed that diagnosis tended to be missed in patients with stage 1 AKI, presence of tumors, or higher serum albumin, while diagnosis tended to be timely in patients with a history of CKD, presence of oliguria, receiving RRT, more failed organs and higher blood urea nitrogen. It is unsatisfactory that only 20.7% of patients with stage 1 AKI were diagnosed in a timely manner, in accordance with the findings of previous studies [18, 19], which revealed that the concept that even a small increase in SCr could increase mortality was not fully comprehended by clinicians. The patients with presence of tumors were also easily misdiagnosed probably due to the concept that a small change in SCr was not as important as the tumors themselves. In addition, patients with higher serum albumin appeared well enough, which may be the reason that clinicians neglected a mild change in SCr. In contrast, those patients with a history of CKD, presence of oliguria, or more severe diseases received sufficient attention, which resulted in a higher diagnosis rate of AKI [18]. Awareness of these characteristics may help to improve the diagnosis rate of AKI.

How to reduce the missed diagnosis rate of AKI presents a great clinical challenge. It is obvious that the missed diagnosis rate is mainly attributed to insufficient recognition of the severity of AKI. However, this situation cannot be changed immediately, but rather by educating and training clinicians. The “0 by 25” initiative of the International Society of Nephrology recommends more frequent serum monitoring in high risk populations, point-of-care testing for SCr, and electronic alerting systems for AKI [20], although a recent study found no improvement in clinical outcomes among hospitalized patients [21]. Based on the results of this study and the experiences from developed countries, an electronic alert system for AKI may be suitable and urgently needed for tertiary hospitals in developed areas of China.

Although our results are informative and add evidence to AKI epidemiology and diagnosis in China, this study has some limitations. First, this was a retrospective observational study in which 30-day mortality was analyzed. However, data was insufficient for analysis of long-term follow-up. Second, a minority of the hospitalized patients were examined for SCr at least two times, thus the incidence of AKI may have been underestimated and the accuracy of recovery of renal function was limited. Third, urinary data were recorded insufficiently, which also contributed to underdiagnosis of AKI. Fourth, this study provided a view of incidence and diagnosis of AKI in a tertiary teaching hospital in southeast China, and could not be generalized to all over the world in which diagnosis or practice patterns may be variable across other populations. Finally, there was a bias between the actual kidney function and baseline kidney function, which was calculated with the minimal SCr level during hospitalization.

## Conclusion

In conclusion, AKI is characterized by a high incidence and high missed diagnosis rate in hospitalized adult patients in this tertiary hospital in a developed area in China. Considering that the missed diagnosis rate was relatively high, interventions for improving the diagnosis of AKI are urgently needed.

## Additional file

Additional file 1: Table S1. Univariate logistic regression analysis on patient prognosis. **Table S2.** Multivariable logistic regression analysis on patient prognosis. **Table S3.** Univariate logistic regression analysis on renal prognosis. **Table S4.** Multivariable logistic regression analysis on renal prognosis. **Table S5.** Univariate logistic regression analysis on failure to timely diagnosis (DOCX 25 kb)

## Abbreviations

AKI: Acute kidney injury
CKD: Chronic kidney injury
eGFR: Estimated glomerular filtration rate
ICU: Intensive care unit
RRT: Renal replacement therapy
SCr: Serum creatinine
