# Supplementary material for: Incidence and diagnosis of Acute kidney injury in hospitalized adult patients: a retrospective observational study in a tertiary teaching Hospital in Southeast China
Source: BMC Nephrol. 2017 Jun 24;18:203. doi: 10.1186/s12882-017-0622-6 (PMC5483286; doi:10.1186/s12882-017-0622-6)
Supplement: Additional file 1: Table S1. — Univariate logistic regression analysis on patient prognosis. Table S2. Multivariable logistic regression analysis on patient prognosis. Table S3. Univariate logistic regression analysis on renal prognosis. Table S4. Multivariable logistic regression analysis on renal prognosis. Table S5. Univariate logistic regression analysis on failure to timely diagnosis (DOCX 25 kb) [file 12882_2017_622_MOESM1_ESM.docx]

**Supplementary Materials**

**Table S1. Univariate logistic regression analysis on patient prognosis**

| Variables | OR | 95% CI | P value |
| --- | --- | --- | --- |
| Age (year) | 1.02 | 1.02-1.03 | <0.001* |
| City | 0.63 | 0.50-0.80 | 0.001* |
| Interval between admission and AKI diagnosed (days) | 1.01 | 1.00-1.02 | 0.012* |
| Diabetes | 1.32 | 1.00-1.75 | 0.051* |
| Chronic lung disease | 2.16 | 1.41-3.30 | <0.001* |
| Chronic liver disease | 1.64 | 1.10-2.44 | 0.016* |
| Chronic kidney disease | 1.00 | 0.67-1.48 | 0.988 |
| Malignancy | 1.33 | 1.05-1.69 | 0.018* |
| Admission eGFR(ml/min/1.73m^2^) | 1.00 | 1.00-1.00 | 0.177 |
| Presence of oliguria | 5.15 | 3.92-6.77 | <0.001* |
| Extra-renal organ failure | 3.10 | 2.65-3.62 | <0.001* |
| Receiving renal replacement therapy | 2.12 | 1.59-2.83 | <0.001* |
| AKI stage 2: stage 1 | 1.75 | 1.28-2.38 | <0.001* |
| AKI stage 3: stage 1 | 3.14 | 2.42-4.08 | <0.001* |
| White blood cells (10^9^/L) | 1.03 | 1.01-1.04 | <0.001* |
| Hemoglobin (g/L) | 0.99 | 0.99-1.00 | 0.002* |
| Platelet count (10^9^/L) | 1.00 | 1.00-1.00 | <0.001* |
| Total bilirubin (µmol/L) | 1.01 | 1.00-1.01 | <0.001* |
| Serum albumin(g/L) | 0.94 | 0.92-0.95 | <0.001* |
| Blood urea nitrogen (mmol/L) | 1.02 | 1.01-1.03 | <0.001* |
| Primary disease |  |  |  |
| Cardiovascular disease | 1.80 | 1.21-2.68 | 0.004* |
| Malignancy | 1.84 | 1.26-2.69 | 0.001* |
| Digestive disease | 1.45 | 0.95-2.21 | 0.083* |
| Cardiovascular disease | 2.96 | 1.76-4.96 | <0.001* |
| Infection | 5.40 | 3.54-8.24 | <0.001* |
| Operation | 0.59 | 0.40-0.88 | 0.009* |
| [Trauma](http://dict.youdao.com/w/trauma/#keyfrom=E2Ctranslation) | 3.05 | 1.30-7.13 | 0.010* |
| [Pulmonary](http://dict.youdao.com/w/pulmonary/#keyfrom=E2Ctranslation) [disease](http://dict.youdao.com/w/disease/#keyfrom=E2Ctranslation) | 7.52 | 4.68-12.06 | <0.001* |
| Number of extra-renal organ failure | | |  |
| 1 | 2.06 | 1.92-3.28 | <0.001* |
| 2 | 12.60 | 8.65-18.36 | <0.001* |
| 3 | 16.92 | 7.27-39.40 | <0.001* |
| 4 or more | 57.17 | 7.46-438.34 | <0.001* |

ACEI/ARB: angiotension conversion enzyme inhibitor/ angiotension II receptor blocker; AKI: acute kidney injury; eGFR: estimated glomerular filtration rate;

**Table S2. Multivariable logistic regression analysis on patient prognosis**

| Variables | 30-day mortality | | |
| --- | --- | --- | --- |
|  | P value | OR | 95%CI |
| Age (year) | <0.001 | 1.05 | 1.03-1.08 |
| Chronic Liver Disease (yes: no) | 0.005 | 14.97 | 2.29-97.64 |
| Presence of oliguria (yes: no) | 0.005 | 3.12 | 1.40-6.92 |
| Extra-Renal Organ Failure (yes: no) | <0.001 | 3.80 | 2.19-6.60 |
| Presence of malignancy (yes: no) | 0.002 | 5.29 | 1.83-15.29 |
| [Pulmonary](http://dict.youdao.com/w/pulmonary/#keyfrom=E2Ctranslation) [Disease](http://dict.youdao.com/w/disease/#keyfrom=E2Ctranslation) (yes: no) | 0.020 | 3.48 | 1.21-9.98 |
| C statistic (95% CI) | 0.90(0.85-0.95) | | |
| [Test](http://dict.youdao.com/w/test/#keyfrom=E2Ctranslation) [of](http://dict.youdao.com/w/of/#keyfrom=E2Ctranslation) [Goodness](http://dict.youdao.com/w/goodness/#keyfrom=E2Ctranslation) [of](http://dict.youdao.com/w/of/#keyfrom=E2Ctranslation) [Fit](http://dict.youdao.com/w/fit/#keyfrom=E2Ctranslation) | χ² =8.151，P=0.419 | | |

**Table S3. Univariate logistic regression analysis on renal prognosis**

| Variables | OR | 95% CI | P value |
| --- | --- | --- | --- |
| Age (year) | 1.02 | 1.01-1.02 | <0.001* |
| City | 0.72 | 0.57-0.92 | 0.008* |
| Interval between admission and AKI diagnosed (days) | 1.00 | 1.00-1.01 | 0.086* |
| Diabetes | 1.18 | 0.89-1.58 | 0.243 |
| Chronic lung disease | 2.04 | 1.33-3.13 | 0.001* |
| Chronic liver disease | 1.22 | 0.81-1.84 | 0.334 |
| Chronic kidney disease | 1.75 | 1.19-2.57 | 0.004* |
| Malignancy | 1.15 | 0.90-1.46 | 0.261 |
| Admission eGFR(ml/min/1.73m^2^) | 0.99 | 0.99-0.99 | <0.001* |
| Presence of oliguria | 7.28 | 5.49-9.65 | <0.001* |
| Extra-renal organ failure | 1.92 | 1.68-2.19 | <0.001* |
| Receiving renal replacement therapy | 7.49 | 5.45-10.29 | <0.001* |
| AKI stage 2: stage 1 | 1.97 | 1.39-2.79 | 0.001* |
| AKI stage 3: stage 1 | 7.16 | 5.35-9.58 | <0.001* |
| White blood cells (10^9^/L) | 1.01 | 1.00-1.02 | 0.032* |
| Hemoglobin (g/L) | 0.99 | 0.98-0.99 | <0.001* |
| Platelet count (10^9^/L) | 1.00 | 1.00-1.00 | 0.022* |
| Total bilirubin (µmol/L) | 1.00 | 1.00-1.00 | <0.001* |
| Serum albumin(g/L) | 0.94 | 0.92-0.96 | <0.001* |
| Blood urea nitrogen (mmol/L) | 1.04 | 1.03-1.05 | <0.001* |
| Etiology of AKI |  |  |  |
| ACEI/ARB | 0.81 | 0.49-1.33 | 0.398 |
| Diuretic | 1.26 | 1.00-1.60 | 0.052* |
| Nephrotoxic antibiotics | 1.48 | 1.16-1.88 | 0.001* |
| [chemotherapeutic](http://dict.youdao.com/w/chemotherapeutic/#keyfrom=E2Ctranslation)  [drugs](http://dict.youdao.com/w/drugs/#keyfrom=E2Ctranslation) | 0.85 | 0.52-1.38 | 0.505 |
| Hypovolemia | 1.38 | 1.10-1.73 | 0.006* |
| Heart failure | 1.09 | 0.85-1.38 | 0.501 |
| Operation | 0.30 | 0.23-0.39 | <0.001* |
| Sepsis | 2.00 | 1.42-2.80 | <0.001* |
| Number of extra-renal organ failure | | |  |
| 1 | 1.35 | 1.04-1.76 | 0.026* |
| 2 | 4.67 | 3.34-6.53 | <0.001* |
| 3 | 5.30 | 2.63-10.67 | <0.001* |
| 4 or more | 19.46 | 4.35-87.06 | <0.001* |

ACEI/ARB: angiotension conversion enzyme inhibitor/ angiotension II receptor blocker; AKI: acute kidney injury; eGFR: estimated glomerular filtration rate;

**Table S4. Multivariable logistic regression analysis on renal prognosis**

| Variables | Renal loss | | |
| --- | --- | --- | --- |
|  | P value | OR | 95%CI |
| Age (year) | 0.001 | 1.01 | 1.00-1.02 |
| AKI stage 2: stage 1 | 0.003 | 1.82 | 1.22-2.69 |
| AKI stage 3: stage 1 | <0.001 | 2.98 | 2.01-4.40 |
| Presence of oliguria (yes: no) | <0.001 | 2.00 | 1.36-2.95 |
| Receiving renal replacement therapy (yes: no) | <0.001 | 2.99 | 1.96-4.56 |
| Extra-renal organ failure (yes: no) | <0.001 | 1.59 | 1.35-2.95 |
| Hemoglobin (g/L) | 0.002 | 0.99 | 0.99-1.00 |
| Total bilirubin (µmol/L) | <0.001 | 1.00 | 1.00-1.01 |
| Serum albumin (g/L) | 0.045 | 0.98 | 0.96-1.00 |
| Operation (yes: no) | <0.001 | 0.39 | 0.28-0.54 |
| C statistic (95% CI) | 0.83(0.81-0.85) | | |
| [Test](http://dict.youdao.com/w/test/#keyfrom=E2Ctranslation) [of](http://dict.youdao.com/w/of/#keyfrom=E2Ctranslation) [Goodness](http://dict.youdao.com/w/goodness/#keyfrom=E2Ctranslation) [of](http://dict.youdao.com/w/of/#keyfrom=E2Ctranslation) [Fit](http://dict.youdao.com/w/fit/#keyfrom=E2Ctranslation) | χ² =7.249，P=0.510 | | |

AKI: acute kidney injury;

**Table S5. Univariate logistic regression analysis on failure to timely diagnosis**

| Variables | OR | 95% CI | P value |
| --- | --- | --- | --- |
| Age (year) | 0.99 | 0.98-0.99 | <0.001* |
| Male: female | 0.97 | 0.78-1.22 | 0.807 |
| City | 0.76 | 0.61-0.96 | 0.020 |
| Interval between admission and AKI diagnosed (days) | 1.01 | 1.00-1.02 | 0.061* |
| Cardiovascular disease | 0.98 | 0.79-1.22 | 0.890 |
| Diabetes | 0.76 | 0.58-1.00 | 0.054 |
| Chronic lung disease | 0.47 | 0.30-0.72 | 0.001* |
| Chronic liver disease | 0.94 | 0.63-1.40 | 0.776 |
| Chronic kidney disease | 0.26 | 0.17-0.40 | <0.001* |
| Malignancy | 1.65 | 1.30-2.10 | <0.001* |
| Admission eGFR(ml/min/1.73m^2^) | 1.03 | 1.03-1.04 | <0.001* |
| Presence of oliguria | 0.08 | 0.06-0.12 | <0.001* |
| Extra-renal organ failure | 0.57 | 0.50-0.65 | <0.001* |
| Receiving renal replacement therapy | 0.02 | 0.01-0.04 | <0.001* |
| AKI stage 2: stage 1 | 0.80 | 0.57-1.12 | 0.187 |
| AKI stage 3: stage 1 | 0.08 | 0.06-0.10 | <0.001* |
| White blood cells (10^9^/L) | 1.00 | 0.99-1.01 | 0.887 |
| Hemoglobin (g/L) | 1.01 | 1.01-1.01 | <0.001* |
| Platelet count (10^9^/L) | 1.00 | 1.00-1.00 | 0.107 |
| Total bilirubin (µmol/L) | 1.00 | 1.00-1.00 | 0.076 |
| Serum albumin(g/L) | 1.07 | 1.05-1.09 | <0.001* |
| Blood urea nitrogen (mmol/L) | 0.92 | 0.90-0.93 | <0.001* |

AKI: acute kidney injury; eGFR: estimated glomerular filtration rate;
